# Can biopsies be omitted after normal colposcopy in women referred with low-grade cervical cytology? A prospective cohort study

**DOI:** 10.1186/s12905-021-01537-5

**Published:** 2021-11-19

**Authors:** Mette Mindedahl Jespersen, Berit Bargum Booth, Lone Kjeld Petersen

**Affiliations:** 1 University of Southern Denmark, SDU, Odense, Denmark; 2 Department of Gynecology and Obstetrics, Randers Regional Hospital, Randers, Denmark; 3 Department of Clinical Medicine, Aarhus University, Aarhus, Denmark; 4 Department of Gynecology and Obstetrics, Odense University Hospital, Odense, Denmark; 5 OPEN Open Patient Data Explorative Network, University of Southern Denmark, Odense, Denmark

**Keywords:** Atypical squamous cells of the cervix, Squamous intraepithelial lesions, Cervical intraepithelial neoplasia, Biopsy, Colposcopy, Diagnosis, Precancerous conditions, Conization

## Abstract

**Background:**

Controversy surrounds whether women with low-risk cytology screening results but a normal colposcopic assessment should have random biopsies taken. The aim of this study was to determine the yield of CIN2+ from one to four cervical biopsies in women with cytology of LSIL or ASCUS and a normal colposcopic impression.

**Methods:**

Between January 2017 and September 2020, women over 18 years old referred for colposcopic examination due to either an abnormal smear (ASCUS+) or follow-up after previous cervical intraepithelial neoplasia (CIN) were invited to participate in the study. All study participants underwent colposcopic examination and had four biopsies taken. The biopsies were analyzed separately.

**Results:**

In total, 1327 women with abnormal cervical cancer screening results or attending follow-up after a previous CIN diagnosis were enrolled in the study and examined by colposcopy. Of these, 173 were newly referred with cytology of LSIL or ASCUS and had a normal colposcopic impression and four adequate biopsies. Of these, 22.0% were diagnosed with CIN2+. When combining the results of the four biopsies, we found a 100% relative increase in CIN2+ cases compared to using only one biopsy (from 11.0% to 22.0%, *P* = 0.006).

**Conclusion:**

As we found CIN2+ from random cervical biopsies in 22.0% of women with cytology of LSIL or ASCUS who had a normal colposcopic impression, we advocate performing four random cervical biopsies at the squamocolumnar junction in such women.

*Trial registration* NCT04249856, January 31 2020 (retrospectively registered).

## Background

Colposcopy is used to determine the optimal biopsy site(s) in women with abnormal cytology or women referred with symptoms such as coital spotting. Sensitivity of colposcopic-directed biopsy (CDB) for cervical intraepithelial neoplasia grade 2 or worse (CIN2+) can be influenced by several factors. Smaller lesions of lower grade [1], age > 50 years and postmenopausal status [2, 3] has been shown to lower the diagnostic consistency between CDB and conization. CDB sensitivity increases when CIN2+ is associated with human papillomavirus (HPV) 16/18 [4, 5] and when the cut-point for defining an abnormal colposcopic impression is lowered [6].

The ability to correctly diagnose cervical dysplasia is positively correlated with the number of biopsies taken [1, 7–9]. Taking four biopsies gives an agreement rate of 95.2% between the worst biopsy diagnosis and the conization diagnosis [10]. However, this result was obtained in a predominantly high-grade squamous intraepithelial lesions (HSIL) population.

Controversy surrounds whether women with abnormal cytology screening results but a normal colposcopic assessment (with a normal-appearing cervix) should have random 4-quadrant biopsies taken.

In Pretorius et al. [6] most of the marginal increase in yield of CIN2+ by random biopsy was seen in women with normal colposcopy (85% (120/141) in Shanxi Province Cervical Cancer Screening Study (SPOCCS) and 57% (20/35) in Shenzhen Cervical Cancer Screening Trial (SHENCCAST)). They concluded that a preferred subset to perform random biopsy is when the colposcopic impression is normal. Also, in SPOCCS a higher proportion of CIN2+ cases were diagnosed by random biopsy in colposcopies associated with cytology other than HSIL or cancer [6].

The Danish national guidelines recommend four biopsies in all women who undergo colposcopy to avoid under-diagnosis of CIN [11]. Conversely, the American Society for Colposcopy and Cervical Pathology (ASCCP) does not recommend non-targeted biopsies for women referred for colposcopy at the lowest level of risk, i.e. less than HSIL cytology, no evidence of HPV 16/18, and a completely normal colposcopic impression (i.e., no acetowhitening, metaplasia or other visible abnormality) [12].

A survey of colposcopists accredited with the British Society of Colposcopy and Cervical Pathology (BSCCP) showed that most colposcopists (56%) aimed to take two biopsies to diagnose CIN. Very few (16.2%) reported taking random biopsies routinely from areas of the cervix that appeared normal [13].

The aim of this study was to determine the yield of CIN2+ from one to four cervical biopsies among women referred to colposcopy for evaluation of cytology of low-grade squamous intraepithelial lesion or atypical squamous cells of undetermined significance (LSIL or ASCUS) cytology in whom the colposcopic impression was normal.

## Methods

This prospective cohort study was conducted at the departments of gynecology and obstetrics at Randers Region Hospital, Horsens Region Hospital, and Aalborg University Hospital, Denmark and at a gynecological private practice in Aarhus, Denmark.

In Denmark, women with abnormal cervical cytology are referred for colposcopy where they have four biopsies taken, regardless of the colposcopic impression. Women may also undergo colposcopy as part of a control program after previous CIN diagnosis [11]. The cytology can be taken as a part of the routine cervical cancer screening program or because of symptoms such as coital spotting. The national guidelines recommend that for women with transformation zone (TZ) type 3 either ECC (endocervical curettage) or an endocervical cytobrush is used. When this study was conducted, HPV-test was not used routinely in Denmark but could be performed as a triage method in women ≥ 30 years old with ASCUS cytology. Screening and subsequently, treatment and follow-up for cervical dysplasia is free of charge in Denmark.

Between January 2017 and September 2020, women over 18 years old referred to colposcopic examination due to either an abnormal smear (ASCUS+) or follow-up after previous CIN were invited to participate in the study. All study participants underwent a colposcopic examination and had four biopsies taken. Exclusion criteria were cervical biopsies taken within the last 6 months, pregnancy within the last 3 months, previous conization, or previous pelvic radiation therapy.

Women included at Randers Regional Hospital and Aalborg University Hospital were examined using a dynamic spectral imaging (DSI) colposcope (DYSIS Medical Ltd., Edinburgh, UK). Women included at Horsens Regional Hospital and the private gynecological clinic in Aarhus were examined using a regular colposcopy (a Leisegang colposcope and an Olympus colposcope, respectively). The colposcopies were performed by nurses, residents, and consultants who routinely performed colposcopies at the facilities. Nurse colposcopists had attended the Comprehensive Colposcopy course by the ASCCP and were personally supervised by consultants for their first 25 procedures.

The biopsies were taken with a 3 mm biopsy forceps then placed in separate vials containing formalin and marked with the corresponding numbers 1–4. Biopsies were taken from the squamocolumnar junction (SCJ) in each cervical quadrant after application of acetic acid (3%). If colposcopy showed discrete signs of visible lesions the first biopsy was taken from the site interpreted as the ‘’worst’’ by the colposcopist. The 2nd biopsy was taken as a DSI-directed biopsy after the DSI-colposcope identified the most suspicious area. If these two biopsy sites were the same, this was noted. The remaining two or three biopsies were taken as either additional biopsies from other visible lesions or as random biopsies from the remaining cervical quadrants. All biopsies were analyzed separately. Biopsies taken at Randers Regional Hospital and Horsens Regional Hospital were analyzed by one of two gynecological histopathologists at Randers Regional Hospital. Biopsies taken at Aalborg University Hospital were analyzed by the Department of Pathology at Aalborg University Hospital. Finally, biopsies taken at the gynecological private practice in Aarhus were analyzed by the Department of Pathology at Aarhus University Hospital.

The colposcopist noted whether the transformation zone was type 1 (fully visible SCJ), type 2 (partially visible), or type 3 (not visible). All participants were subsequently managed clinically according to the national guidelines [11].

The following information was collected at inclusion via a questionnaire completed by the participants: age, height, weight, smoking habits, parity, contraception, and HPV vaccination status. Information on referral cytology and previous CIN diagnosis was obtained from medical records.

### A normal colposcopic impression

In this study a normal colposcopic impression was based on the clinical assessment by the colposcopist. The colposcopists were asked to evaluate whether the colposcopic impression was thought to be normal, low-grade or high-grade and to note any visible changes (i.e. acetowhitening, atypical vessels, punctuation and/or mosaic changes). Discrete colposcopic observations may represent normal findings according to several colposcopic indices [14, 15]. Therefore, women were included if the colposcopist assessed the overall colposcopic impression to be normal. We also performed analysis on a subgroup of women with a completely normal colposcopic impression, where not even faint visible changes, thought to be normal by the colposcopist, was accepted. All women had four cervical biopsies taken.

### Statistical analysis

Histopathological diagnoses were categorized into the following categories: No dysplasia (including inflammation and unspecific reactive changes), CIN grade 1, and CIN2+ (CIN grade 2 or worse, including ungradable CIN). When analyzing all four cervical biopsies together, the worst histological diagnosis in any of the four biopsies was considered the worst grade of dysplasia present. Percentage agreement between the histological diagnosis of four biopsies and cone specimen and the relative increase in CIN2+ cases when comparing one, two, three and four biopsies was calculated. A *P* value ≤ 0.05 was considered statistically significant.

We used STATA 16.0 analytic software (STATA Corp, LP, College Station, TX) for the statistical analysis.

## Results

**Fig. 1:**
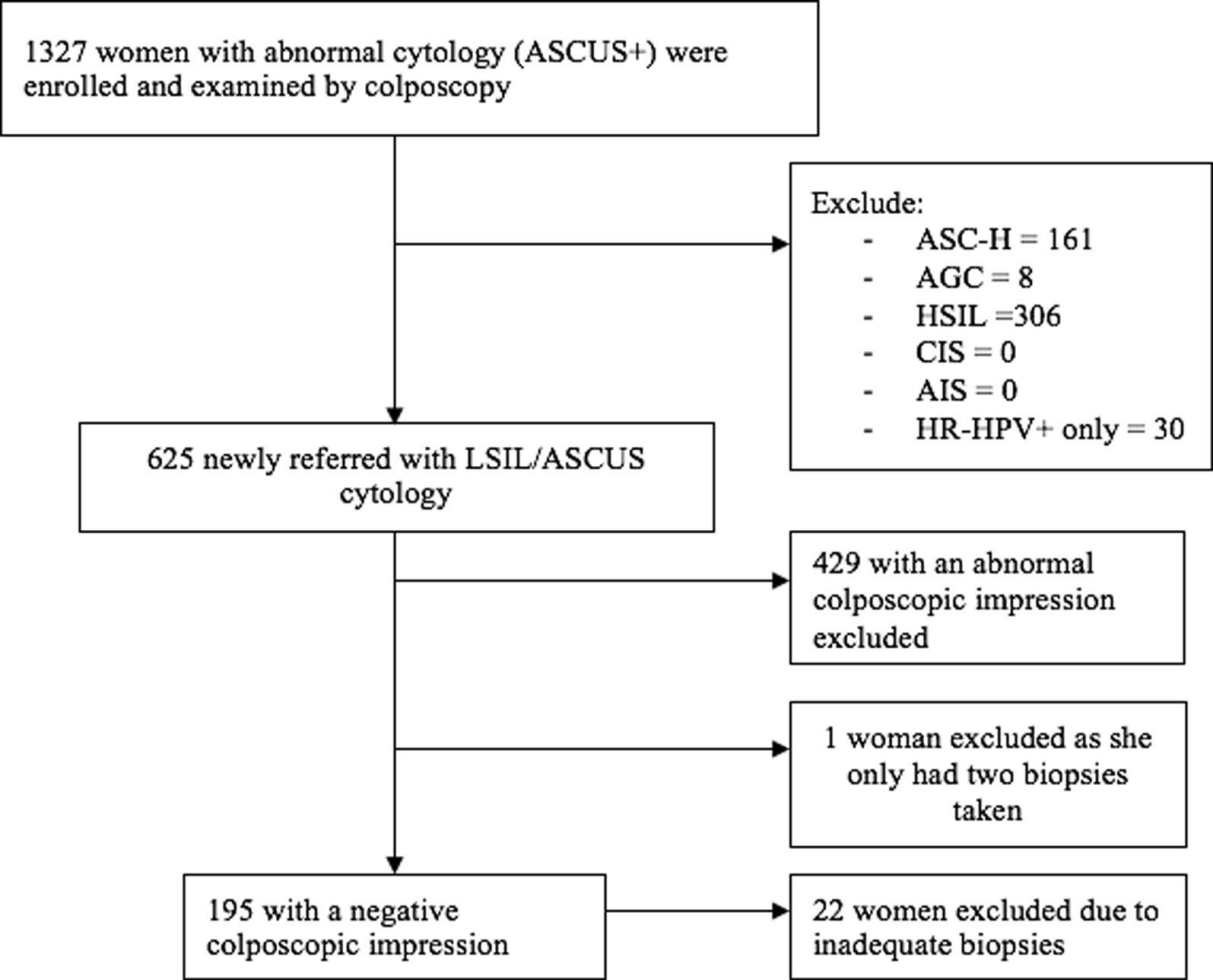
Participant flow diagram. LSIL, low-grade squamous intraepithelial lesion; ASCUS, atypical squamous cells of undetermined significance; ASC-H, atypical squamous cells—cannot exclude HSIL; AGC, atypical glandular cells; HSIL, high-grade squamous intraepithelial lesion; CIS, carcinoma in situ; AIS, adenocarcinoma in situ; HR-HPV, high-risk HPV

A total of 1327 women with either abnormal cervical cancer screening results or attending follow-up after previous CIN were enrolled in the study and examined by colposcopy. Of these, 625 were newly referred with LSIL or ASCUS cytology, 196 were assessed to have a normal colposcopic impression. Women who did not have four adequate biopsies taken were excluded, leaving 173 newly referred women with cytology of LSIL or ASCUS and a normal colposcopic impression for further analysis (Fig. 1). Data were analyzed for histological diagnosis and clinical factors such as age, menopause status, and visibility of the cervical transformation zone.

The median age of the included women was 33.2 years (range 20–79). There were 109 (63.0%) cases referred with ASCUS, while 64 were referred with LSIL (37.0%) (Table 1). Of these, 52.6% (91/173) had a HPV-test done. Among the women referred with cytology of ASCUS, 87.2% (68/78) were hr-HPV positive while 100% (13/13) with LSIL were hr-HPV positive. None of the women had ECC performed. Twenty-two percent [95% CI 16.0–28.9] (38/173) were diagnosed with CIN2+ (Table 2). In this study, 137 women (79.2%) had a completely normal colposcopic impression and 36 (20.8%) had transparent acetowhitening, discrete vessel changes, fine punctuations and/or mosaic changes of the cervix. However, the colposcopic impression was still assessed to be normal by the colposcopist and therefore they were included in our main investigation. Among the women with discrete colposcopic findings, assessed to be normal by the colposcopist, 44.4% (16/36) were diagnosed with CIN2+. When excluding women with any recorded visible changes of the cervix, 16.1% [95% CI 10.4–23.3] (22/137) were diagnosed with CIN2+ (n = 137) (Table 2).

**Table 1:** Selected characteristics of the colposcopies performed

Education level of colposcopist	Nurse	51 (29.5%)
	Resident	55 (31.8%)
	Consultant	67 (38.7%)
Facility site	Randers Region Hospital	78 (45.1%)
	Horsens Region Hospital	65 (37.6%)
	Aalborg University Hospital	2 (1.2%)
	Gynecological private practice	28 (16.2%)
Referral diagnosis	ASCUS	109 (63.0%)
	LSIL	64 (37.0%)
HPV type determined	Yes	91 (52.6%)
	No	82 (47.4%)
HPV type	HPV 16	5 (5.5%)
	HPV 18	1 (1.1%)
	HPV other high risk	69 (75.8%)
	HPV 16 and other high risk	3 (3.3%)
	HPV 18 and other high risk	2 (2.2%)
	HPV 16, 18 and other high risk	1 (1.1%)
	HPV tested – no HPV determined	10 (11.0%)
Former history of cervical dysplasia	Yes	55 (31.8%)
	No	118 (68.2%)
Visibility of the Squamocolumnar junction	Yes (type 1)	100 (57.8%)
	Partially (type 2)	26 (15.0%)
	No (type 3)	47 (27.2%)
Median age		33.2 years (range 20–79)
Vaccination status	Vaccinated	95 (53.8%)
	Not vaccinated	73 (42.2%)
	Ongoing	3 (1.7%)
	Undisclosed	4 (2.3%)

HPV, Human papillomavirus

**Table 2:** Correlation between the number of biopsies and the diagnosis of CIN2+ for all women, women without any visible changes of the cervix and women who underwent LEEP

Diagnosis	Biopsy 1		Biopsy 1 + 2		Biopsy 1 + 2 + 3		Biopsy 1 + 2 + 3 + 4	
	n	% (95%CI)	n	% (95%CI)	n	% (95%CI)	n	% (95%CI)
*All included women (n* = *173)*								
No dysplasia	121	69.9 (62.5–76.7)	102	59.0 (51.2–66.4)	91	52.6 (44.9–60.2)	76	43.9 (36.4–51.7)
CIN1	33	19.1 (13.5–25.7)	44	25.4 (19.1–32.6)	52	30.1 (23.3–37.5)	59	34.1 (27.1–41.7)
CIN2+	19	11.0 (6.7–16.6)	27	15.6 (10.5–21.9)	30	17.3 (12.0–23.8)	38	22.0 (16.0–28.9)
*Women without any visible changes of the cervix (n* = *137)*								
No dysplasia	100	73.0 (64.7–80.2)	85	62.0 (53.4–70.2)	76	55.5 (46.7–64.0)	65	47.4 (38.9–56.1)
CIN1	27	19.7 (13.4–27.4)	37	27.0 (19.8–35.3)	45	32.8 (25.1–41.4)	50	36.5 (28.4–45.1)
CIN2+	10	7.3 (3.6–13.0)	15	10.9 (6.3–17.4)	16	11.7 (6.8–18.3)	22	16.1 (10.3–23.3)
*Women who underwent LEEP (n* = *21)*								
No dysplasia	5	23.8 (8.2–47.2)	3	14.3 (3.0–36.3)	3	14.3 (3.0–36.3)	0	0.0 (0–16.1)
CIN1	6	28.6 (11.3–52.2)	4	19.0 (5.4–41.9)	3	14.3 (3.0–36.3)	3	14.3 (3.0–36.3)
CIN2+	10	47.6 (25.7–70.2)	14	66.7 (43.0–85.4)	15	71.4 (47.8–88.7)	18	85.7 (63.7–97.0)

CIN1, Cervical intraepithelial neoplasia grade 1; CIN2+, cervical intraepithelial neoplasia grade 2 or worse; LEEP, loop electrosurgical excision procedure

When only one biopsy result was used, 11.0% (19/173) of the women were diagnosed with CIN2+. When two biopsies were used, 15.6% (27/173) of the women were diagnosed with CIN2+. This was a relative increase of 41.8% in detection of CIN2+ (*P* = 0.2). When combining the first three biopsies, there was a relative increase of 57.3% in detection of CIN2+ compared to taking just one biopsy (from 11.0 to 17.3% (30/173), *P* = 0.09). When combining all four biopsies, we found a 100% relative increase in detection of CIN2+ compared to taking one biopsy (from 11.0% to 22.0% (38/173), *P* = 0.006) (Table 2).

Of the 173 included women, 47 had type 3 TZ, i.e. the SCJ was not visible (27.2%). In more than 50% of cases, however, both endocervical and ectocervical cells were represented in the biopsies (ranging from 53.2 to 59.6% in the four biopsies). When the SCJ was not represented in the biopsies, the histopathologists identified only ectocervix in more than 94% of cases (ranging from 94.7 to 100% in the four biopsies). For the women with type 2 TZ the SCJ was represented in more than 69% of cases (ranging from 69.2% to 92.3% in the four biopsies) and for the women with type 1 TZ the SCJ was represented in more than 83% of cases (ranging from 83 to 87% in the four biopsies). Only 2.1% (1/47) of the women with a type 3 TZ were diagnosed with CIN2+ while 11.5% (3/26) with type 2 TZ (partially visible SCJ) and 34.0% (34/100) with type 1 TZ (fully visible SCJ) were diagnosed with CIN2+ (Table 3).

**Table 3:** comparison of the yield in CIN2+ from random biopsies in women with the three different types of transformation zones

Yield in CIN2+ from random biopsies in women with three different transformations zones								
Diagnosis	Biopsy 1		Biopsy 1 + 2		Biopsy 1 + 2 + 3		Biopsy 1 + 2 + 3 + 4	
	n	% (95%CI)	n	% (95%CI)	n	% (95%CI)	n	% (95%CI)
TZ type 1 (n = 100)	17	17.0 (10.2–25.8)	25	25.0 (16.9–34.7)	28	28.0 (19.5–37.9)	34	34.0 (24.8–44.2)
TZ type 2 (n = 26)	2	7.7 (0.9–25.1)	2	7.7 (0.9–25.1)	2	7.7 (0.9–25.1)	3	11.5 (2.4–30.2)
TZ type 3 (n = 47)	0	0 (0–0)	0	0 (0–0)	0	0 (0–0)	1	2.1 (0.1–11.3)

Of the 173 included women, 21 (12.1%) underwent loop electrosurgical excision procedure (LEEP). The agreement between the worst histological biopsy diagnosis (of any four) and the final histological diagnosis based on the LEEP specimen was 85.7% (95% CI 63.7–97.0) (Table 2). The histological diagnosis of the biopsies underestimated the CIN-grade compared with the LEEP diagnosis in 4.8% of cases (95% CI 0.12–23.8), but biopsies overestimated (or removed) the CIN grade in 9.5% of cases (95% CI 1.2–30.4) (Table 4). Using the first biopsy result, 47.6% (10/21) of the women who underwent LEEP were diagnosed with CIN2+. When comparing this to the agreement between all four biopsies (18/21, 85.7%) it was a relative increase of 80.0% in the detection of CIN2+ (*P* = 0.009) (Table 2).

**Table 4:** Agreement between diagnosis based on the four biopsies and diagnosis based on the LEEP specimen (n = 21)

Diagnosis based on all four biopsies	Worst diagnosis of LEEP specimen				Agreement between biopsies and LEEP	
	No dysplasia	CIN1	CIN2+	Total	n	% (95% CI)
CIN1	1	1	1	3 (14.3%)	18	85.7 (63.7–97.0)
CIN2+	0	1	17	18 (85.7%)		
Total	1 (4.8%)	2 (9.5%)	18 (85.7%)	21 (100%)		

## Discussion

In our study population of newly referred women with cytology of LSIL or ASCUS and a normal colposcopic impression, we found that approximately one out of five women were diagnosed with CIN2+ when the results of all four biopsies were taken into account. We found a relative increase of 100% in the detection of CIN2+ when using four biopsies compared to using one biopsy result (from 11.0% to 22.0%, *P* = 0.006).

Twenty-one of our study participants underwent LEEP. Compared against the final histological diagnosis of the LEEP specimen, the four random biopsies combined correctly identified the CIN-grade in 85.7% of cases. A previous study investigated the accuracy of CDB in women with cytology of LSIL or ASCUS and minor colposcopic findings. The CDB was immediately followed by LEEP (n = 68). Thirty-one (45.6%) of the biopsies accurately detected the disease severity [16]. This indicates a possible increase in diagnostic accuracy when taking several biopsies.

In the ALTS study [17], no difference was found in the risk of CIN2+ within 2 years of follow-up between women with histological CIN1 and women with no CIN at initial colposcopy, suggesting that lesions go unrecognized. ALTS demonstrated that reliance on just a single biopsy for diagnosis may lead to underestimation of the true CIN-grade. Our results are concordant with ALTS and demonstrate that the risk of underestimating disease severity is substantial in this group of women. The SUCCEED trial analyzed biopsies in women referred for LEEP because of CIN3 detected in a previous biopsy [18]. Before performing the LEEP, the colposcopist marked the worst appearing and a normal appearing area on the cervical surface with ink. Substantial discrepancies between expected and observed histology were observed where 30% of the expected CIN3 lesions were CIN1 or normal, and 40% of the expected normal specimens were CIN2+. This demonstrates that identifying the worst lesion on the cervix can be challenging, even in a previously confirmed CIN3 population. Such pathologic discrepancies between CDB and LEEP have also been reported in other studies [19–21]. This is concordant with our results where 44.4% CIN2+ cases was diagnosed in women with discrete colposcopic findings assessed to be normal by the colposcopist.

An explanation why CIN2+ lesions in women referred with low-grade cytology are not found at colposcopy may be inter-observer variability in the colposcopic interpretation in the CIN-grade [17, 22] which may result in underdiagnosis of prevalent CIN2+. Also, Massad et al. found poor correlation between colposcopic impression and biopsy histology and that the negative predictive value of a benign colposcopic impression was only 38% for women who had a biopsy taken [23]. Furthermore, lesion size may influence the colposcopic accuracy. CIN2+ lesions that are detected from cytology of LSIL or ASCUS have been shown to be smaller than those detected from HSIL cytology [24–26].

The women included in our study were assessed to have a normal colposcopy, and even though this group of women is considered to have the lowest risk, we found that 22.0% had CIN2+ when taking four biopsies. When only one biopsy result was used, 11.0% were diagnosed with CIN2+. Only 50% of CIN2+ cases was found in the first biopsy, suggesting that taking several random biopsies increases diagnostic accuracy for CIN2+ in women with cytology of LSIL or ASCUS and a normal colposcopic impression.

Our findings suggest that non-targeted biopsies can be an important strategy to improve the management of women at the lowest risk of CIN. The benefits of detecting clinically significant disease that could become a cervical cancer is apparent. However, it is important to consider that some CIN2+ lesions may regress without treatment. It has been argued that small CIN2+ lesions not seen at initial colposcopy might not be clinically relevant and will be diagnosed at later rounds of screening if they do not regress. The TOMBOLA trial found that the risk of high-grade CIN within 3 years after a normal colposcopy was sufficiently low for women referred with low-grade cytology to justify return to 3-year yearly recall [27]. However, currently there is no way to separate lesions that will regress on their own and lesions that persists. Taking multiple biopsies will enhance our ability to make a suitable follow-up plan for the individual woman if needed.

This study was conducted in Denmark where screening, treatment, and follow-up for cervical dysplasia is free of charge. To reduce the cost of pathology when taking multiple biopsies, the biopsies can be submitted in the same vial. However, the pathologist still needs to analyze the additional biopsies.

We chose to include women with type 3 TZ in our investigation (n = 47, 27.2%). The colposcopic impression for these women was assessed to be normal. However, the colposcopic examination was not optimal as the SCJ was not fully visible. Yet, we found that the SCJ was represented in the biopsies in more than 50% of the cases. However, for women with type 1 or 2 TZ the SCJ was represented in more than 69% of cases. The yield of CIN2+ was lower for those with type 3 TZ than for those with type 1 or 2 TZ (2.1% (1/47) and 29.4% (37/126), respectively). We did not perform ECC on any of the women in this study but an explanation for the lower yield of CIN2+ in women with type 3 TZ could be that CIN2+ in women with type 3 TZ is located within the endocervical canal and not easily detected by random biopsies. While our results indicate that performing random biopsies is not as efficient in women with type 3 TZ the biopsies did represent the SCJ in more than 50% of cases. This could especially be of relevance in postmenopausal women with type 3 TZ who do not want a diagnostic conization. In Denmark, the general practice is to take four blind biopsies when the TZ is not visible. If the biopsies are not representative of the SCJ a diagnostic conization is considered in consultation with the women [28].

The ASCCP recommends taking biopsies when there is any degree of visible abnormality present in women with low-risk cytology [12]. According to colposcopic indices [14, 15] discrete colposcopic observations can be considered normal findings. Therefore, we chose to include women based on the colposcopic assessment. However, when excluding all women with any visible changes of the cervix approximately half of the remaining women had dysplasia and one out of six were diagnosed with CIN2+ when taking four biopsies. Confirming, that lesions go unrecognized. Among the women with discrete colposcopic findings, assessed to be normal by the colposcopist, 44.4% were diagnosed with CIN2+. This supports the ASCCP’s recommendation of taking biopsies when there is any degree of visible abnormality.

Taking four biopsies is a key strength of our study as it has been shown to give a high agreement rate between biopsy diagnosis and conization diagnosis. The four biopsies were analyzed separately by histopathologists. Therefore, the women acted as their own controls, and we could compare the individual women’s diagnosis when taking just one biopsy or when combining the subsequent biopsies. Another strength of our study is that it is a prospective study design, reflecting the real clinical setting in Denmark. A limitation of our study is the risk of verification bias. For ethical reasons, women with biopsies that showed no dysplasia did not undergo LEEP, so these women might not have been truly negative. However, verification bias was limited by taking four biopsies from each woman. Although the number of women in our study was relatively small, our findings indicate that biopsies should not be omitted in women referred with low-grade cervical cytology. Furthermore, our results demonstrate that the number of diagnosed CIN2+ cases increase when the results of four random biopsies are used as opposed to one biopsy.

## Conclusion

In a study population of women referred with cytology of LSIL or ASCUS and a normal colposcopic impression, we found that approximately one out of five women were diagnosed with CIN2+ based on the results of four biopsies. The detection of CIN2+ was doubled when using the results of four biopsies compared to using one biopsy. This leads us to recommend taking four random biopsies in women referred for colposcopic evaluation with cytology of LSIL or ASCUS when the colposcopic impression is normal.

## Data Availability

Restrictions apply to the availability of these data, which were used under the license of this study. Data are available from the authors upon reasonable request and with the permission from the Danish Data Protection Agency.

## Abbreviations

TZ: Transformation zone
HSIL: High-grade squamous intraepithelial lesion
CIN: Cervical intraepithelial neoplasia
ASCCP: American Society for Colposcopy and Cervical Pathology
HPV: Human papillomavirus
BSCCP: British Society of Colposcopy and Cervical Pathology
LSIL: Low-grade squamous intraepithelial lesion
ASCUS: Atypical squamous cells of undetermined significance
CIN2+: Cervical intraepithelial neoplasia grade 2 or worse
CIN3+: Cervical intraepithelial neoplasia grade 3 or worse
SCJ: Squamous columnar junction
LEEP: Loop electrosurgical excision procedure
ASC-H: Atypical squamous cells – cannot exclude HSIL
AGC: Atypical glandular cells
CIS: Carcinoma in situ
AIS: Adenocarcinoma in situ
HR-HPV: High-risk HPV
CIN1: Cervical intraepithelial neoplasia grade 1
CDB: Colposcopy-directed biopsy
DSI: Dynamic spectral imaging
ECC: Endocervical curettage
SPOCCS: Shanxi Province Cervical Cancer Screening Study
SHENCCAST: Shenzhen Cervical Cancer Screening Trial
